# In Vitro Study of Licorice on IL-1β-Induced Chondrocytes and In Silico Approach for Osteoarthritis

**DOI:** 10.3390/ph14121337

**Published:** 2021-12-20

**Authors:** Akhtar Ali, YoungJoon Park, Jeonghoon Lee, Hyo-Jin An, Jong-Sik Jin, Jong-Hyun Lee, Jaeki Chang, Dong-Keun Kim, Bonhyuk Goo, Yeon Cheol Park, Kang-Hyun Leem, Shin Seong, Wonnam Kim

**Affiliations:** 1 Cnh Center for Cancer Research, Cnh Corporation, Gangnam-gu, Seoul 06154, Korea; akhtar.ali@cnhgroup.kr (A.A.); yjparkcb@cnhgroup.kr (Y.P.); 2 Herbal Crop Research Division, National Institute of Horticultural and Herbal Science, Rural Development Administration, Eumseong 27709, Chungcheongbuk-do, Korea; artemisia@korea.kr; 3 Department of Pharmacology, College of Korean Medicine, Sangji University, Wonju 26339, Gangwon-do, Korea; sangjipharm@gmail.com; 4 Department of Oriental Medicine Resources, Jeonbuk National University, Iksan 54596, Jeollabuk-do, Korea; jongsik.jin@jbnu.ac.kr (J.-S.J.); kdk5796@daum.net (D.-K.K.); 5 Department of Natural Medicine, College of Pharmacy, Dongduk Women’s University, Seongbuk-gu, Seoul 02748, Korea; naturalmed@dongduk.ac.kr; 6 Crop Production & Physiology Division, National Institute of Crop Science, Rural Development Administration, Wanju 55365, Jeollabuk-do, Korea; changjk@korea.kr; 7 Department of Acupuncture & Moxibustion, Kyung Hee University Hospital at Gangdong, Gangdong-gu, Seoul 05278, Korea; goossi9@khu.ac.kr; 8 Department of Acupuncture & Moxibustion Medicine, College of Korean Medicine, Kyung Hee University, Dongdaemun-gu, Seoul 02447, Korea; icarus08@khu.ac.kr; 9 Division of Pharmacology, College of Korean Medicine, Semyung University, Jecheon 27136, Chungcheongbuk-do, Korea; lkh@semyung.ac.kr; 10 Soram Korean Medicine Hospital, Gangnam-gu, Seoul 06154, Korea; ss9335@soramhospital.kr

**Keywords:** osteoarthritis, chondrocyte hypertrophy, licorice, Wongam

## Abstract

Osteoarthritis (OA) is a common degenerative joint disorder that affects joint function, mobility, and pain. The release of proinflammatory cytokines stimulates matrix metalloproteinases (MMPs) and aggrecanase production which further induces articular cartilage degradation. Hypertrophy-like changes in chondrocytes are considered to be an important feature of OA pathogenesis. A *Glycyrrhiza* new variety, Wongam (WG), was developed by the Korea Rural Development Administration to enhance the cultivation and quality of Glycyrrhizae Radix et Rhizoma (licorice). This study examined the regulatory effect of WG against hypertrophy-like changes such as RUNX2, Collagen X, VEGFA, MMP-13 induction, and Collagen II reduction induced by IL-1β in SW1353 human chondrocytes. Additionally, in silico methods were performed to identify active compounds in licorice to target chondrocyte hypertrophy-related proteins. WG showed inhibitory effects against IL-1β-induced chondrocyte hypertrophy by regulating both HDAC4 activation via the PTH1R/PKA/PP2A pathway and the SOX9/β-catenin signaling pathway. In silico analysis demonstrated that 21 active compounds from licorice have binding potential with 11 targets related to chondrocyte hypertrophy. Further molecular docking analysis and in vivo studies elicited four compounds. Based on HPLC, isoliquiritigenin and its precursors were identified and quantified. Taken together, WG is a potential therapeutic agent for chondrocyte hypertrophy-like changes in OA.

## 1. Introduction

Osteoarthritis (OA), also referred to as degenerative joint disorder, is caused by multiple factors including age, sex, obesity, genetics, joint injury, and diabetes [1]. OA symptoms limit patients’ quality of life due to their significant impact on joint function, mobility, and pain [2]. OA is characterized by pathological changes such as the irreversible deterioration of the articular cartilage, inflammation of the synovial membrane, damage of the subchondral bone, and osteophyte formation [3]. In recent years, studies have suggested that the inflammatory and fibrotic changes of infrapatellar fat pad and the pathological changes of meniscus have important roles in OA pathology [4,5]. The release of proinflammatory cytokines such as interleukin 6 (IL-6), interleukin 1β (IL-1β), and tumor necrosis factor α (TNF-α) stimulates matrix metalloproteinases (MMPs) and aggrecanase production which further degrade the articular cartilage matrix particularly collagen type II and aggrecan in OA [6]. Chondrocyte hypertrophy-like changes have been increasingly recognized as an important feature of OA pathogenesis [7]. Hypertrophic chondrocytes, relevant to the terminal differentiation of chondrocytes, express elevated levels of type X collagen (Collagen X), runt-related transcription factor 2 (RUNX2), and MMP13, whereas markers for hyaline cartilage, such as type II collagen (Collagen II) and SRY-related high-mobility group-box gene 9 (SOX9) decrease in OA conditions [8]. Several signaling pathways such as Wnt/β-Catenin, Indian hedgehog (Ihh), parathyroid hormone-related peptide (PTHrP), transforming growth factor-beta (TGF-β), fibroblast growth factor (FGF), SOX9, and bone morphogenetic protein (BMP) signaling are involved in hypertrophy-like changes in chondrocytes [9].

Glycyrrhizae Radix et Rhizoma (licorice) is one of the most frequently used medicinal plants which is used to treat hepatitis, gastritis, gastric ulcers, bronchitis, influenza, and arthritis in traditional herbal medicine [10]. Many studies report the anti-inflammatory, anti-oxidative, anti-fibrosis, anti-viral, anti-depressant, and anti-cancer effects of licorice [11]. Licorice contains bioactive components such as triterpenoid saponins, flavonoids, phenolic compounds, coumarins, and essential oils which exhibit its pharmacological activities [12]. A novel variety of *Glycyrrhiza*, named Wongam (WG), was selected through the hybridization method, *Glycyrrhiza glabra* × *Glycyrrhiza uralensis*, by the Korea Rural Development Administration [13]. In addition, the content rate of glycyrrhizin and liquiritigenin in WG is 3.96% and 0.8%, respectively, which meets the Korean Pharmacopoeia Standards [14]. Additionally, WG has shown improved root growth, yield, brown spot resistance, and lodging compared to *Glycyrrhiza uralensis* [14]. Previous studies have reported the immunomodulatory, anti-allergic, anti-oxidative, anti-cancer, anti-inflammatory, and anti-neuroinflammatory effects of WG [15,16,17,18,19]. 

In the present study, we investigated the underlying molecular mechanism regulated by WG on hypertrophy-like changes in IL-1β-induced SW1353 chondrocytes. An in silico-based systems approach was performed to identify active compounds related to the molecules that inhibit chondrocyte hypertrophy. Furthermore, we wanted to validate the major active compounds that have the potential to interact with the important proteins in the pathogenesis of OA. To achieve this goal, active compounds were validated from studies using surgically induced OA in vivo models, and compound–target docking analysis was performed. To the best of our knowledge, our investigation is the first to report the therapeutic potential of WG and its major active components against chondrocyte hypertrophy and OA.

## 2. Results

### 2.1. Chemical Characterization of WG by High-Performance Liquid Chromatograph (HPLC) System

**Figure 1:**
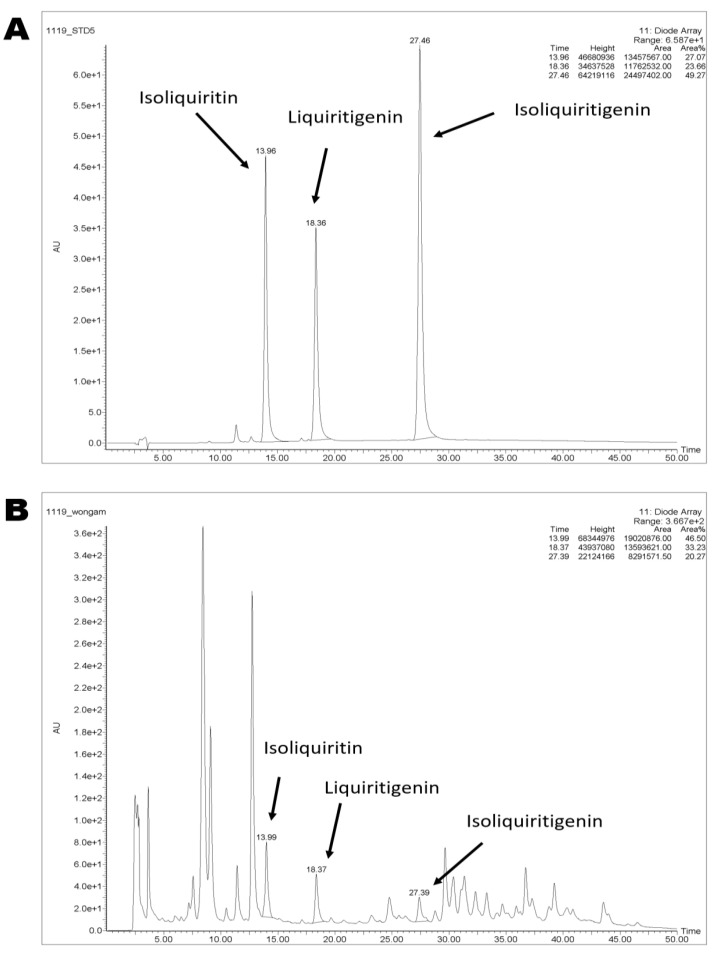
HPLC chromatograms of standard (**A**) isoliquiritin, liquiritigenin, and isoliquiritigenin. HPLC chromatograms of (**B**) WG extract.

Flavonoid compounds which were reported as components of licorice and picked up in this study were analyzed. We analyzed and quantified three compounds, isoliquiritin, liquiritigenin, and isoliquiritigenin. Retention times were 13.9, 18.3, and 27.4 min, respectively (Figure 1). Additionally, the amount of isoliquiritin, liquiritigenin, and isoliquiritigenin was 1.41, 1.15, and 0.33 mg/g in WG. Furthermore, when WG extract was analyzed with LC-QTOF-MS, kaempferol, kaempferol glycosides, kaempferol derivatives, quercetin glycosides, and quercetin derivatives were identified (Tables S1 and S2).

### 2.2. WG Ameliorated IL-1β Induced Chondrocyte Hypertrophy-like Changes

**Figure 2:**
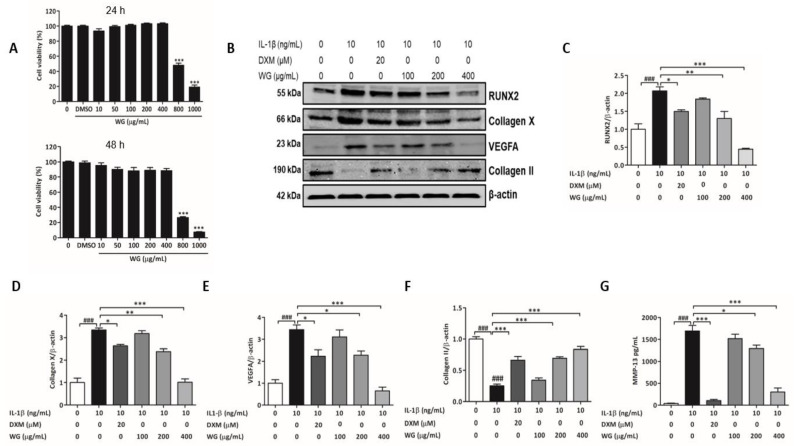
Cytotoxicity and effects of WG against IL-1β-induced hypertrophic changes. (**A**) The cytotoxicity of WG on chondrocytes was determined at different concentrations using MTT assay. (**B**) Protein expression and quantitative analysis of (**C**) RUNX2, (**D**) Collagen X, (**E**) VEGFA, and (**F**) Collagen II expression relative to β-actin. (**G**) MMP-13 production was determined by ELISA. SW1353 cells were pretreated with indicated concentration of WG or DXM for 4 h followed by IL-1β for 24 h. Data shown represent mean ± SEM (*n* = 3). (### *p* < 0.001, vs. control; * *p* < 0.05, ** *p* < 0.01, and *** *p* < 0.001 vs. IL-1β).

SW1353 chondrocytes were incubated with different concentrations (0, 10, 50, 100, 200, 400, 800, and 1000 μg/mL) of WG for 24 and 48 h. Results from the MTT assay showed that WG (800 and 1000 μg/mL) significantly decreased cell viability, whereas no cytotoxicity was observed up to 400 μg/mL (Figure 2A). Based on these data, all subsequent experiments were conducted using concentrations of 100–400 μg/mL. Studies have suggested that, at an appropriate concentration, dexamethasone (DXM) exhibits chondroprotective effects [20,21]. DXM (20 μM) was included in the study as a positive control.

Hypertrophic differentiation of chondrocytes is characterized by the increased expression of RUNX2, Collagen X, VEGFA, and MMP-13 and the decreased expression of cartilage-specific markers, such as Collagen II and SOX9 [8,22,23]. WG treatment significantly suppressed the IL-1β-induced increase in RUNX2 expression at 200 (0.65-fold) and 400 μg/mL (0.22-fold) in SW1353 cells (Figure 2B,C). WG, dose dependently, inhibited Collagen X expression by 0.72-fold (at 200 µg/mL) and 0.3-fold (at 400 µg/mL) and also VEGFA expression by 0.68-fold (at 200 µg/mL) and 0.19-fold (at 400 µg/mL) compared to IL-1β-treated cells (Figure 2B,D,E). Whereas, WG ameliorated the IL-1β-induced suppression of Collagen II in SW1353 cells (Figure 2B,F). Next, we checked the effect of WG on MMP13, an ECM-degrading enzyme, which is also highly expressed in hypertrophic chondrocytes in OA [7,8]. ELISA results showed that MMP-13 levels significantly increased by IL1-β (42-fold) compared to the control (Figure 2G). WG significantly downregulated MMP-13 production by 0.76-fold (at 200 µg/mL) and 0.18-fold (at 400 µg/mL), respectively, compared to IL-1β-treated cells (Figure 2G).

### 2.3. WG Stimulated HDAC4 Nuclear Translocation via PKA and PP2A

**Figure 3:**
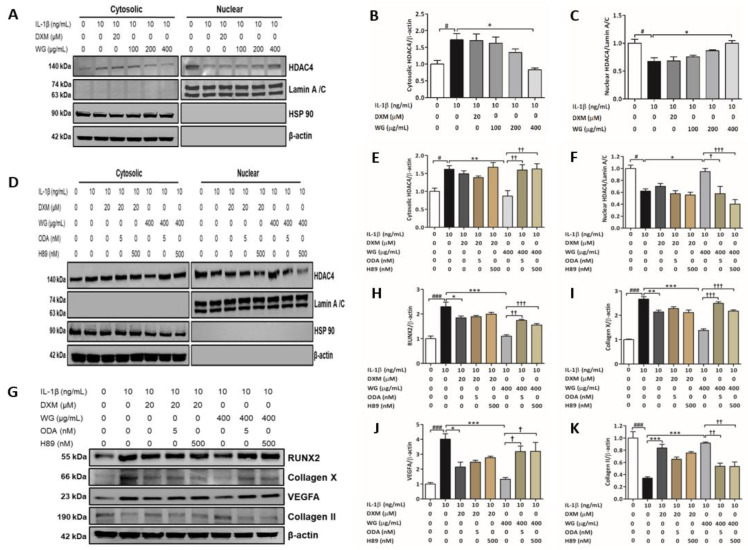
Effect of WG on HDAC4 nuclear translocation. (**A**) Western blot analysis of cytosolic and nuclear fractions using HDAC4 antibody, (**B**) quantitative analysis of cytosolic HDAC4 relative β-actin, (**C**) quantitative analysis of nuclear HDAC4 relative to Lamin A/C. SW1353 cells were pre-treated with indicated concentration of WG or DXM for 4 h in 0.2% BSA media followed by IL-1β for 24 h. (**D**) Western blot analysis of cytosolic and nuclear fractions using HDAC4 antibody, (**E**) quantitative analysis of cytosolic HDAC4 relative β-actin, (**F**) quantitative analysis of nuclear HDAC4 relative to Lamin A/C. (**G**) Protein expression and quantitative analysis of (**H**) RUNX2, (**I**) Collagen X, (**J**) VEGFA, and (**K**) Collagen II expression relative to β-actin. SW1353 cells were pre-treated with ODA or H89 in 0.2% BSA media, or cell media was either changed to 0.2% BSA media for 1 h and then treated with WG or DXM for 4 h followed by IL-1β for 24 h. Data shown represent mean ± SEM (*n* = 3). (# *p* < 0.05 and ### *p* < 0.001 vs. control; * *p* < 0.05, ** *p* < 0.01, and *** *p* < 0.001 vs. IL-1β; † *p* < 0.05, †† *p* < 0.01, and ††† *p* < 0.001 vs. WG).

HDAC4 has been reported to repress chondrocytes hypertrophy by regulating RUNX2 expression [24]. We found that IL-1β significantly decreased nuclear HDAC4 levels, and WG treatment dose dependently increased nuclear HDAC4 levels (Figure 3A,C). While WG induced HDAC4 translocation from the cytoplasm to the nucleus, the maximum effect was achieved at 400 μg/mL (1.47-fold) against IL-1β-treated cells (Figure 3A–C). Interestingly, DXM treatment had no effect on HDAC4 translocation (Figure 3A,C).

In chondrocytes, the cyclic adenosine monophosphate (cAMP)/protein kinase A (PKA) cascade potentiates HDCA4 localization to the nucleus by activating protein phosphatase 2A (PP2A) [25]. Our results showed that the WG-induced nuclear translocation was decreased by the PKA inhibitor H89 (0.42-fold) and as well as the PP2A inhibitor okadiac acid (ODA) (0.61-fold) (Figure 3D–F). This suggests that WG-upregulated HDAC4 translocation is mediated by the PKA/PP2A signaling pathway (Figure 3D–F). Next, we asked the question about whether the inhibitory effects of WG on RUNX2 expression are regulated by HDAC4 via the PKA/PPA2 pathway. Our data showed that ODA and H89 treatment increased RUNX2 (1.58-fold, 1.42-fold), Collagen X (1.81-fold, 1.58-fold), VEGFA (2.41-fold, 2.42-fold), respectively, whereas ODA and H89 treatment decreased Collagen II (0.59-fold, 0.58-fold), respectively, compared to WG-treated SW1353 cells (Figure 3G–K).

### 2.4. PTH1R Mediated HDAC4 Activation by WG

**Figure 4:**
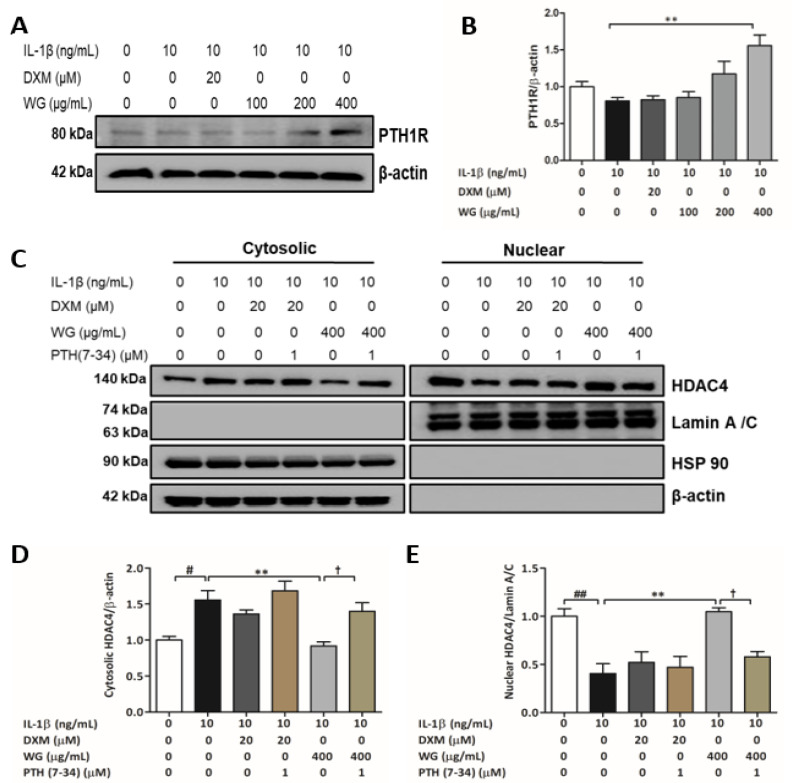
Effect of WG on PTH1R. (**A**) Western blot analysis of PTH1R. (**B**) Quantitative analysis of PTH1R relative to β-actin. SW1353 cells were pre-treated with indicated concentration of WG or DXM for 4 h in 0.2% BSA media followed by IL-1β for 24 h. (**C**) Western blot analysis of cytosolic and nuclear fractions using HDAC4 antibody, (**D**) quantitative analysis of cytosolic HDAC4 relative β-actin, (**E**) quantitative analysis of nuclear HDAC4 relative to Lamin A/C. SW1353 cells were pre-treated with WG or DXM for 4 h in 0.2% BSA media in the presence or absence of PTH (7–34) followed by IL-1β for 24 h. (# *p* < 0.05 and ## *p* < 0.01 vs. control; ** *p* < 0.01 vs. IL-1β; † *p* < 0.05 vs. WG).

Interestingly, we found that WG treatment significantly increased parathyroid hormone 1 receptor (PTH1R) expression (1.93-fold), but DXM treatment did not (Figure 4A,B). Studies have reported that PTHrP binds to PTH1R and causes a delay in chondrocyte hypertrophy via HDAC4-mediated RUNX2 suppression [25,26,27]. To examine whether the increase in nuclear HDAC4 by WG was affected by PTHrP/PTH1R signaling, we used recombinant human PTH (7–34), which is a PTH1R antagonist. As expected, the HDAC4 nuclear translocation induced by WG (2.95-fold) was significantly inhibited by PTH (7–34) treatment (0.55-fold) (Figure 4C–E).

### 2.5. WG Increased SOX9 Expression and Decreased β-Catenin Activation

**Figure 5:**
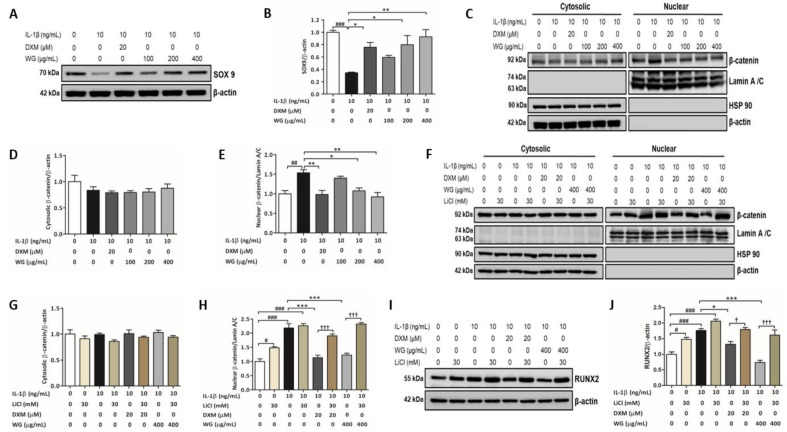
WG increased SOX9 expression and attenuated the IL-1β-induced β-catenin signaling pathway. (**A**) Western blot analysis of SOX9. (**B**) Quantitative analysis of SOX9 relative to β-actin. (**C**) Western blot analysis of cytosolic and nuclear and fractions using β-catenin antibody, quantitative analysis of (**D**) cytosolic β-catenin relative to β-actin and (**E**) nuclear β-catenin relative to Lamin A/C. SW1353 cells were pre-treated with indicated concentration of WG or DXM for 4 h in 0.2% BSA media followed by IL-1β for 24 h. (**F**) Western blot analysis of cytosolic and nuclear and fractions using β-catenin antibody, quantitative analysis of (**G**) cytosolic β-catenin relative to β-actin and (**H**) nuclear β-catenin relative to Lamin A/C. (**I**) Western blot analysis of RUNX2, (**J**) quantitative analysis of RUNX2 relative to β-actin. SW1353 cells were pre-treated with WG or DXM for 4 h in 0.2% BSA media followed by IL-1β for 24 h in the presence or absence of LiCl. Data shown represent mean ± SEM (*n* = 3). (# *p* < 0.05, ## *p* < 0.01 and ### *p* < 0.001 vs. control; * *p* < 0.05, ** *p* < 0.01, and *** *p* < 0.001 vs. IL-1β; † *p* < 0.05 and ††† *p* < 0.001 vs. DXM or WG).

Studies indicate that SOX9 can regulate chondrocyte hypertrophy by blocking RUNX2 activation and also by interacting with β-catenin signaling [28,29]. WG treatment significantly increased SOX9 expression by 2.3-fold (at 200 µg/mL) and 2.7-fold (at 400 µg/mL) compared to IL-1β-treated cells (Figure 5A,B). The increased nuclear levels of β-catenin in IL-1β-treated cells were reduced significantly by a WG 200 (0.65-fold) and 400 µg/mL (0.60-fold) treatment, respectively (Figure 5C,D). Lithium chloride (LiCl), a β-catenin agonist, was used to further confirm the effects of WG on β-catenin inhibition. Co-treatment with LiCl upregulated the nuclear translocation of β-catenin against the downregulated nuclear β-catenin levels by WG (Figure 5E,F). Moreover, LiCl co-treatment reversed the inhibitory effects of WG on RUNX2 expression (Figure 5G,H).

### 2.6. Computational Network Analysis to Identify Licorice Compounds Related to Chondrocyte Hypertrophy

**Figure 6:**
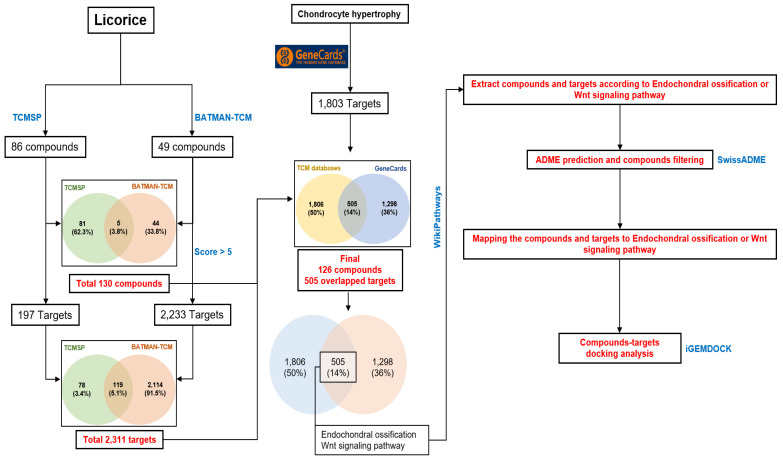
Flow work in silico pharmacology network analysis.

Computational network analysis was applied to understand the pharmacologic effect of licorice compounds on chondrocyte hypertrophic changes. First, compound and target information of licorice were downloaded from the TCMSP and BATMAN-TCM databases. As a result, a total of 86 and 43 compounds were extracted from TCMSP and BATMAN-TCM, respectively (Figure 6). For each of the 86 and 43 compounds, the associated 197 and 2233 targets were extracted, respectively, followed by the merging of each extracted compound and target databases (Figure 6 and Tables S3 and S4). A total of 130 compounds and 2311 targets were identified (Figure 6). GeneCards database was acquired to find out targets related to chondrocyte hypertrophy. In this work, we obtained 1803 targets from GeneCards and merged them with the 2311 targets from the TCMSP and BATMAN-TCM databases. Finally, a total of 505 targets and 126 compounds were extracted against licorice and chondrocyte hypertrophy (Figure 6). To identify the biological mechanisms of the 505 targets, we performed gene enrichment analysis with the WikiPathways database. As a result, the 505 targets were significantly enriched to 141 unique pathways after Bonferroni corrections (Table S6). Interestingly, among the 141 pathways, endochondral ossification (*p*-value: 2.04 × 10^−12^) and the Wnt signaling pathway (*p*-value: 9.16 × 10^−7^) which are strongly related to chondrocyte hypertrophy were significantly enriched (Table S6).

### 2.7. Validation of Major Active Compounds in Licorice Based on the Experimental OA Model

**Figure 7:**
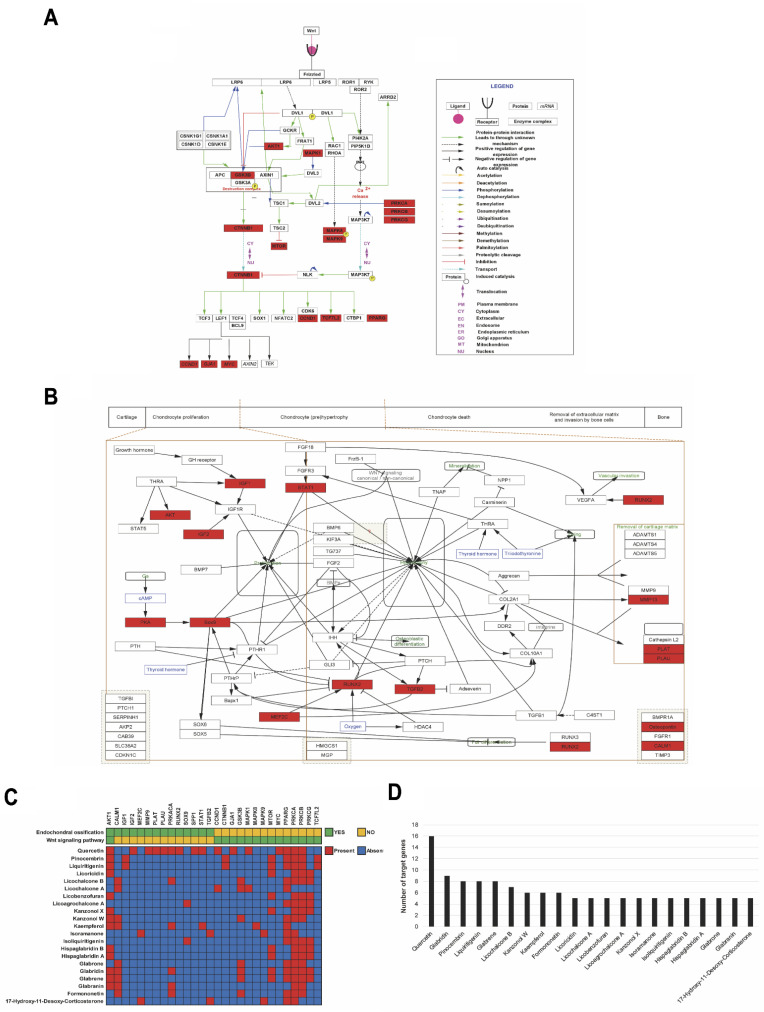
The 21 active compounds of licorice and its targets in (**A**) Wnt-signaling pathway and (**B**) endochondral ossification. (**C**) Representation of 21 active compounds and their targets (red: targeted and black: non-targeted). (**D**) The number of targeted genes in Wnt-signaling pathway or endochondral ossification according to 21 active compounds.

To identify key active compounds in licorice for chondrocyte hypertrophy, we focused on endochondral ossification and the Wnt signaling pathway from WikiPathways. The compounds which have more than five targets involved in endochondral ossification or the Wnt signaling pathway were extracted (Tables S7 and S8). As a result, a total of 23 compounds were selected (Table S8). The absorption, distribution, metabolism, and elimination (ADME) profiling were performed against the 23 compounds using SwissADME to evaluate their pharmacotherapeutic potentials. We considered parameters such as gastrointestinal (GI) absorption, bioavailability score, and cLOGP to evaluate the drug-likeness of the 23 compounds in ADME filtering (detailed information is available in Section 4) (Table S8). As a result, except for l-aspartic acid and 5-hydroxyisophthalic acid, a total of 21 compounds passed the ADME filtering (cLOGP > 1, GI absorption = High and Bioavailability score >0.55) and were considered as active compounds (Table 1). The l-aspartic acid and 5-hydroxyisophthalic acid had a low GI absorption probability and low cLOGP value (cLOGP < 1), respectively (Table 1). Among the 117 genes which are related to endochondral ossification or the Wnt signaling pathway (Table S7), 28 genes were associated with the 21 active compounds (Figure 7). Among the 21 active compounds, quercetin targeted 16 genes including RUNX2 and protein kinase cAMP-activated catalytic subunit alpha (PRKACA), which targeted the majority of endochondral ossification or the Wnt signaling pathway-related genes (Figure 7B,C). Liquiritigenin and pinocembrin targeted eight genes including catenin beta 1 (CTNNB1) and transcription factor 7 like 2 (TCF7L2) (Figure 7B,C). Isoliquiritigenin and licoagrochalcone A targeted five genes including SOX9 (Figure 7B,C). PRKACA was targeted by various active compounds including licochalcone B, formononetin, glabridin, glabranin, kaempferol, and quercetin (Figure 7B).

**Figure 8:**
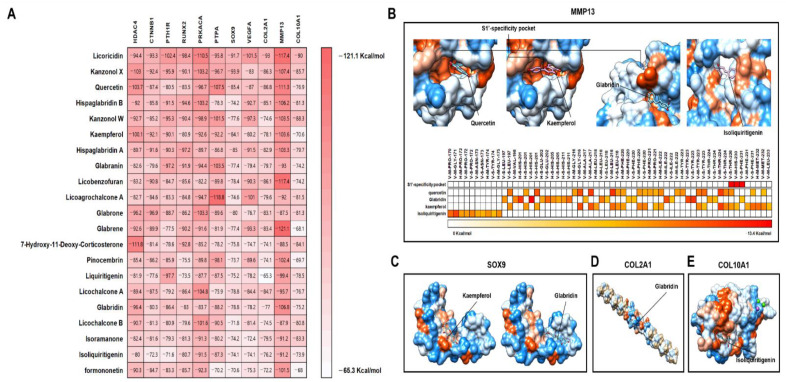
Result of protein docking analysis between 21 active compounds and 11 main proteins. (**A**) Representation of docking energy. Represented 3D molecular docking between four active compounds which were reported to OA in previous studies with (**B**) MMP13, (**C**) SOX9, (**D**) COL2A1, and (**E**) COL10A1.

Finally, we performed protein–compound docking analysis between the 21 active compounds and the 11 major proteins which were experimentally validated for chondrocyte hypertrophy (Figure 8A). As a result, all docking energy values between the 21 active compounds and the 11 major proteins were below −65.3 Kcal/mol (Figure 8A). This indicates that the 21 active compounds have the potential to bind stably to the 11 major chondrocyte hypertrophy-related genes (Table S10). Among the 21 active compounds, licoricidin showed the lowest docking energy with PTH1R, RUNX2, PRKACA, VEGFA, and COL2A (Figure 8A). Recent studies in vivo have suggested that quercetin, kaempferol, glabridin, and isoliquiritigenin possess therapeutic effectiveness for OA [30,31,32,33]. Quercetin, kaempferol, and glabridin deeply interacted with the S1’-specificity pocket of MMP-13 (Figure 8B). Kaempferol, glabridin, and isoliquiritigenin have the potential to interact with important proteins in the pathogenesis of OA such as MMP-13, SOX9, COL2A1, and COL10A1 (Figure 8B–E).

## 3. Discussion

In recent years, OA has been steadily rising due to the rapidly aging population [34]. Although there are adverse effects, non-steroidal anti-inflammatory drugs (NSAIDs) have been important treatments for OA. However, these drugs only provide temporary relief from clinical symptoms and fail to prevent the progression of OA [35,36]. Therefore, the identification of pharmacological compounds from natural products that reverse the progression of OA and have minimal side effects will be a potentially attractive approach to develop more effective therapeutics for OA. Licorice has been widely used in traditional herbal medicine due to its pharmacological activities such anti-oxidative, anti-inflammatory, antiviral, anti-cancer, anti-diabetic, hepatoprotective, and cardioprotective activities [37]. Glycyrrhizin, a major component of the licorice plant, has recently been shown to have a protective effect in OA [38]. The Korea Rural Development Administration developed WG, a hybrid between *Glycyrrhiza glabra* × *Glycyrrhiza uralensis* [13]. In the present study, we investigated the effects of WG on IL-1β-induced chondrocyte hypertrophy-like changes in SW1353 cells that play a crucial role in OA pathogenesis. Additionally, the active compounds of licorice for chondrocyte hypertrophy in OA cartilage were validated via a computational network approach.

RUNX2 is a key regulator of chondrocyte hypertrophy, which plays a role in OA development and is involved in the increase of Collagen X, VEGFA, and MMP-13 expression [39,40,41]. Collagen X is produced by hypertrophic chondrocytes [42]. MMP-13 is a matrix metalloproteinase that degrades Collagen II [43]. VEGFA, a well-known angiogenic factor expressed by hypertrophic chondrocytes, is required for chondrocytes survival, cartilage angiogenesis, and endochondral bone development [44]. All of these factors are considered as important biological markers for chondrocytes hypertrophy. In the current study, hypertrophy markers including the RUNX2, Collagen X, VEGFA, and MMP-13 levels which were significantly elevated following IL-1β stimulation were downregulated by WG treatment (Figure 2B–E,G). Whereas, WG induced Collagen II expression inhibited by IL-1β (Figure 2B,F). Hence, according to the results, WG exhibited protective effects against IL-1β-induced chondrocytes by alleviating the hypertrophic transition.

HDAC4 emerged as a central regulator for chondrocyte hypertrophy by inhibiting RUNX2 [45]. In vitro, IL-1β has been demonstrated as inhibiting HDAC4 nuclear translocation [46,47]. We investigated whether WG regulated HDAC4 translocation against IL-1β stimulation. Our data showed that WG increased nuclear HDAC4 expression in IL-1β-induced SW1353 cells. Studies have suggested that cAMP/PKA cascade induces HDCA4 nuclear translocation by activating PP2A in chondrocytes [25,48]. We next determined whether the WG-mediated increase in nuclear HDAC4 levels was associated with the PKA/PP2A pathway. WG mediated the nuclear translocation of HDAC4 that was downregulated by H89 (PKA inhibitor) and okadiac acid (PP2A antagonist) treatment which eventually resulted in the upregulation of hypertrophy markers such as RUNX2, Collagen X, and VEGFA. Interestingly, we found that WG significantly increased the expression of PTH1R (Figure 4A,B). Studies showed that chondrocytes hypertrophy was inhibited by PTHrP signaling through PTH1R, which induced HDAC4 localization to the nucleus via cAMP/PKA and PP2A activation [25,27]. To clarify whether WG induced HDAC4 nuclear translocation via PTH1R signaling, we treated SW1353 cells with PTH (7–34), a PTH1R antagonist. PTH (7–34) treatment attenuated the WG-induced nuclear translocation of HDAC4, suggesting that WG inhibited chondrocyte hypertrophy via PTH1R signaling (Figure 4C–E). Previous reports have shown the anti-inflammatory and protective effects of DXM against cartilage degradation in experimental OA models [20,49,50,51]. Although, DXM significantly suppressed hypertrophy-like changes such as RUNX2, Collagen X, VEGFA, MMP-13 upregulation, and Collagen II downregulation against IL-1β-induced SW1353 cells (Figure 2B–G). In the study, we could not find a significant effect of DXM on HDAC4 nuclear translocation through PTH1R-mediated cAMP/PKA and PP2A signaling (Figure 2 and Figure 3). However, DXM treatment increased SOX9 and decreased β-catenin expression (Figure 5A–D). These finding suggest that DXM might target other signaling pathways that require further investigation.

SOX9 is an important transcription factor that mediates the differentiation of bone marrow mesenchymal stem cells into chondrocytes [52]. Recently, SOX9 has been proposed as being necessary to inhibit hypertrophy in chondrocytes [53]. Studies have reported that β-catenin is involved in the pathogenesis of OA via regulating chondrocytes hypertrophy [54,55]. As a key factor in the Wnt/β-catenin signaling pathway, the expression level of β-catenin in the nucleus directly reflects the activation level of this signaling pathway [56]. Additionally, reports suggested that activated β-catenin enhanced RUNX2 expression in chondrocytes [57,58]. In our study, WG treatment enhanced SOX9 expression whereas the nuclear β-catenin expression was inhibited against IL-1β-induced SW1353 cells (Figure 5A–E). We further explored whether LiCl-mediated β-catenin activation counteracted the inhibitory effect of WG on IL-1β-induced chondrocyte hypertrophy. We observed that LiCl treatment enhanced β-catenin nuclear levels and RUNX2 expression against WG treatment (Figure 5F–J). These results suggest that both SOX9 and β-catenin signaling were associated with the effects of WG against chondrocyte hypertrophy.

The surgically induced animal OA models well reflect the pathophysiological and structural changes in human OA [59]. The anterior cruciate ligament transection (ACLT) and destabilization of the medial meniscus (DMM) models have been proven to induce surgical instability for OA development [60]. Surgically induced heterozygous knockout Runx2+/− mice showed decreased cartilage destruction and osteophyte development by reducing Collagen X and MMP-13 expression [61]. Thus, the pathological process in surgically induced OA models showed RUNX2 mediated chondrocyte hypertrophy-like changes [61]. We conducted a systems computational approach to predict the pharmacological actions of active compounds from licorice. In our results, among the 21 active compounds, four compounds (quercetin, glabridin, isoliquiritigenin, and kaempferol) were experimentally validated with surgically induced OA animal models in previous studies [30,31,32,33]. Quercetin is a flavonoid compound that exhibits anti-proliferative, anti-oxidative, and anti-arthritic effects [62,63,64,65]. Quercetin increased tissue inhibitors of metalloproteinases-1 and superoxide dismutase and decreased MMP-13 expression which attenuated the progression of OA through inhibiting oxidative stress and cartilage degradation [30]. Glabridin is a flavonoid compound that has been reported for its pharmacological activity such as antioxidant, anti-cancer, anti-osteoporotic, anti-inflammatory, and antimicrobial effects [66]. In an ACLT-induced OA model in rats, glabridin inhibited MMP-13 and Adamts5 expression whereas it increased Collagen II and SOX9 expression from the OA cartilage [31]. Glabridin prevented the apoptosis of human chondrocytes from oxidative stress by inducing autophagy in an mTOR-dependent manner [31]. Isoliquiritigenin is a flavonoid compound that exerts anti-cancer, anti-inflammatory, anti-diabetic, hepatoprotective, and cardioprotective effects [67]. Isoliquiritigenin attenuated cartilage destruction in ACLT-induce OA mice by downregulating the Collagen X and MMP-13 levels [32]. Additionally, isoliquiritigenin prevented abnormal bone formation and angiogenesis that lead to OA progression by inhibiting TGF- β release in ACLT mice [32]. Kaempferol is a flavonoid compound that shows biological functions such as antioxidant, anti-inflammatory, anti-cancer, antiallergic, and osteoprotective effects [68]. Kaempferol or co-treatment with apigenin showed therapeutic effects against ACLT-induced OA rats by suppressing TNF-α and IL-1β levels, key factors of OA progression, that resulted in the downregulation of MMP-13 and MMP-3 levels and the upregulation of SOX9 and Collagen II levels [33].

The docking energies between the four active compounds and 11 major targets against chondrocyte hypertrophy were at least less than −60 kcal/mol (Figure 8A). This indicates that the four compounds within licorice may stably bind with the 11 chondrocyte hypertrophy-related targets. Most especially, among the main 11 targets, MMP-13 is a critical therapeutic target for OA progression. In a previous study, two selective non-chelating inhibitors were discovered that attenuated cartilage damage without side effects such as joint fibroplasia [69]. The two inhibitors interacted with the S1’-specificity pocket of MMP-13 with residues including Leu-197, Tyr-223, 225, Gly-227, and Phe-231 (Figure 8B). These residues are keys for determining selective binding property. This indicates that the three active compounds including quercetin, kaempferol, and glabridin may have a binding potential with MMP-13, which may be considered to reduce the progression of OA by directly interacting with the S1’-specificity pocket of MMP-13.

Our data from HPLC analysis showed that among four compounds only isoliquiritigenin were detected and quantified in WG extract (Figure 1). Further LC-QTOF-MS analysis identified kaempferol, kaempferol glycosides, kaempferol derivatives, quercetin glycosides, and quercetin derivatives (Tables S1 and S2). Interestingly, recent studies suggest that isoliquiritin, liquiritigenin, quercetin glycosides, and kaempferol glycosides, as precursors, may convert to isoliquiritigenin, quercetin, and kaempferol, respectively. By hydrolysis, isoliquiritin, a glycoside of isoliquiritigenin, is biotransformed into isoliquiritigenin [70]. After ingestion, ring cleavage of liquiritigenin into isoliquiritigenin occurred by gut bacteria such as *Eubacterium ramulus* [71]. Sequential conversion of rutin, a quercetin glycoside, into quercetin-3-glucoside by *Enterobacteriaceae* and into quercetin by *Lachnospiraceae* [72]. The *Lactobacillus paracasei* A221 strain converted kaempferol-glucosides into kaempferol [73]. Moreover, licorice is a plant-originated natural resource, and many factors involving seed, cultivation, harvest time, and extraction solvents may vary the chemical composition of licorice [74,75,76,77]. Other than small molecules, macromolecules such as polysaccharide may also be the active components of WG. Many studies have reported the biological activities of licorice polysaccharide [78,79,80]. Further study is needed to fully understand the active components in WG that synergistically act on chondrocyte hypertrophy related OA. However, hypertrophy involves morphologic changes such as enlargement of cells with an increase in apoptotic rate [81]. An in vivo study with morphological evidence may have strengthened our results.

## 4. Materials and Methods

### 4.1. Reagents

Dulbecco’s modified Eagle medium (DMEM) and Penicillin-streptomycin from Welgene (Namcheon-myeon, Gyeongsangbuk-do, Korea). Fetal bovine serum (FBS) from Gibco (Carlsbad, CA, USA). Dimethyl sulfoxide (DMSO), 3-(4,5-dimethylthiazol-2-yl)-2,5-diphenyltetrazoliumbromide (MTT), dexamethasone, and lithium chloride from Sigma-Aldrich (St. Louis, MO, USA). Recombinant Human IL-1β from PeproTech (Cranbury, NJ, USA). H89 (#9844) and Okadiac acid (#5934) from Cell Signaling Technology (Bervely, MA, USA). Parathyroid Hormone (7-34), Human Recombinant (228-11343-2) from RayBiotech (Peachtree Corners, GA, USA). Antibodies against VEGFA (ab46154, 1:1000) and Collagen X (ab182563, 1:1000) from Abcam (Cambridge, UK). RUNX2 (#12566, 1:1000), HDAC4 (#5392, 1:1000), SOX9 (#82630, 1:1000), β-catenin (#8480, 1:1000), Lamin A/C (#4777, 1:2000), HRP linked anti-mouse IgG (#7076, 1:5000), and HRP linked anti-rabbit IgG (#7074, 1:5000) from Cell Signaling Technology (Bervely, MA, USA). β-actin (A5411, 1:5000) from Sigma Aldrich (St. Louis, MO, USA). Antibody Collagen II (sc-52658, 1:1000) and PTH/PTHrP-R (sc-12722, 1:1000) from Santa Cruz Biotechnology (Dallas, TX, USA). HSP90 (13171-1-1AP, 1:2500) from Proteintech (Rosemont, IL, USA).

### 4.2. Preparation of WG

The Korea Rural Development Administration provided WG. WG was separately boiled in 2 L of 30% EtOH for 2 h in 100 °C followed by filtration and evaporated by a rotary evaporator. The extraction yield of lyophilized WG extract was 6%. The voucher specimens (WG: BON20028.WG301) were deposited at the herbarium of Korean Medicine at Semyung University.

### 4.3. HPLC and Preparation of Standard and Sample Solutions

Chromatography was performed using a Waters 2695 system (Waters, MA, USA). Detailed conditions were as follows: column, Spusil 5 μm C18-EP column (4.6 × 250 mm, 5 μm, DiKMA, Foothill Ranch, CA, USA); temperature, 35 °C; wavelength, 254 nm; injection volume, 20 μL, flow rate, 1.0 mL/min, mobile phase, distilled water (solvent system A), and acetonitrile (solvent system B) in a gradient mode (solvent B 20% to 100% for 50 min). The stock solution of isoliquiritin, liquiritigenin, and isoliquiritigenin was prepared in methanol. Sample powder (0.5 g) was extracted with 5 mL methanol by means of sonication at room temperature for 1 h. The extracts were then filtered through a syringe filter (0.45 µm).

### 4.4. Cell Culture and Cytotoxicity Assay

The human chondrosarcoma cell line SW1353 from American Type Culture Collection (Manassas, VA, USA). The cells were cultured in DMEM supplemented with 10% FBS and 1% Penicillin-streptomycin at 37 °C and 5% CO_2_ in a humidified incubator.

SW1353 cells, density of 1 × 10^4^ cells/well, were seeded in a 96-well plate followed by incubation for 24 h. After 24 h, the cells were treated with different concentrations of WG for an additional 24 and 48 h. Media were changed to media with MTT dye, and the cells were incubated for another 4 h. After the supernatants were removed, 100 μL of DMSO were added to the cells to dissolve the formazan. Finally, the plate was gently agitated on a shaker, and the absorbance was measured with a microplate reader at 570 nm (Biotek, VT, USA). 

### 4.5. MMP-13 Assay

SW1353 cells, density of 2 × 10^4^ cells/well, were seeded in a 24-well plate and incubated for 24h. The cells were pretreated with different concentrations of WG or DXM for 4 h in DMEM with 0.2% BSA, followed by adding 1ng/mL of IL-1β for an additional 24 h. The media from each well were collected and centrifuged at 1500 rpm for 10 min (4 °C). The MMP-13 levels were measured using the Human MMP-13 ELISA kit from RayBiotech (Peachtree Corners, GA, USA).

### 4.6. Western Blotting Assay

SW1353 cells were extracted using RIPA lysis buffer (0.1% SDS, 1% Triton X-100, 0.5% sodium deoxycholate, 150 mM NaCl, 50 mM Tris HCL (pH 7.4), 2 mM EDTA) supplemented with protease inhibitor cocktail on ice for 10 min. After centrifugation at 14,000× *g* for 30 min (4 °C), the supernatants were collected. The NE-PER nuclear and cytoplasmic protein extraction kit from Thermo Fisher Scientific (Rockford, IL, USA) was used to extract nuclear and cytoplasmic protein. The protein concentrations were determined using Bio-Rad protein assay from Bio-Rad (Hercules, CA, USA). Cell proteins were separated by SDS-PAGE and transferred to nitrocellulose membranes. Transferred immunoblots were blocked with 5% skim milk and then probed with the specific primary antibodies overnight (4 °C). After washing, the membrane was probed with HRP-conjugated secondary antibodies at RT for 1 h. After subsequent washing with TBST, the membrane was developed using ECL reagent from Biomax (Seoul, Korea) and band intensity was quantified with Image J.

### 4.7. Data Collection

Compounds–targets information of Licorice from TCMSP (https://tcmsp-e.com/, accessed on 24 June 2021) [82] and BATMAN-TCM (http://bionet.ncpsb.org.cn/batman-tcm/, accessed on 24 June 2021) [83] databases were downloaded to evaluate system pharmacological effects. In total, 86 and 43 compounds were retrieved from TCMSP and BATMAN-TCM which included 197 and 2233 targets, respectively (Tables S3 and S4). We used UniProt (https://www.uniprot.org/, accessed on 24 June 2021) [84] to convert protein name to gene symbol in TCMSP database. In BATMAN-TCM, compounds and targets with prediction scores less than 5 were excluded. To extract chondrocyte hypertrophy-related genes, we downloaded from GeneCards (https://www.genecards.org/, accessed on 25 June 2021) [85] database by searching with “chondrocyte hypertrophy” as a keyword. In total, 1803 targets were retrieved as chondrocyte hypertrophy-related genes from GeneCards (Table S5).

### 4.8. Gene Ontology Analysis

We performed gene enrichment analysis with WikiPathways (https://www.wikipathways.org/index.php/WikiPathways, accessed on 6 July 2021) by using ClueGO (Version 2.5.8) from Cytoscape (Version 3.8.2) to identify biological mechanisms [86,87,88]. For each pathway, the threshold for proportion and number of associated genes were set above 30% and 10, respectively. In addition, the Bonferroni adjustment was applied for the multiple comparisons problem (Adjusted *p* < 0.05).

### 4.9. ADME Prediction

We performed analysis with ADME by SwissADME web-based tool (http://www.swissadme.ch/, accessed on 15 July 2021) to evaluate the ADME properties of compounds [89]. The GI absorption indicates the prediction of passive absorption probability by gastrointestinal tract [89]. The bioavailability score indicates the probability of 10% oral bioavailability with rats or Caco-2 permeability [89]. To evaluate cellular membrane passive permeability of each compound, we considered consensus LOGP (cLOGP), average values of five LOGP including iLOGP, XLOGP, WLOGP, MLOGP, and SILICOS-IT, as a parameter of lipophilicity with threshold >1 according to previous study for prediction of cellular membrane permeability [89,90].

### 4.10. Protein-Compound Docking Analysis

The SDF and PDB files for compounds and targets were downloaded from PubChem (https://pubchem.ncbi.nlm.nih.gov/, accessed on 16 July 2021) and Protein Data Bank (https://www.rcsb.org/, accessed on 17 July 2021) [91,92]. The solvent, ligand, and unwanted chains were removed from downloaded PDB files using UCSF Chimera software (https://www.cgl.ucsf.edu/chimera/, accessed on 19 July 2021) (Version 1.15, Windows) [93]. We used iGEMDOCK software (http://gemdock.life.nctu.edu.tw/dock/igemdock.php, accessed on 19 July 2021) (Version 2.1, Windows) to perform the compounds–targets docking analysis [94]. We performed docking analysis between compounds and targets with a “Quick Docking” option with population size 150, generations 70, and number of solutions 1.

### 4.11. Statistical Analysis

Data are representative of three independent values and presented as mean ± SEM. Statistical analysis was performed by ANOVA followed by Tukey’s multiple comparison test using GraphPad Prism 5.01 (San Diego, CA, USA). The differences were indicated as statistically significant when *p* < 0.05.

## 5. Conclusions

In conclusion, WG regulated the hypertrophic change, such as an increase in RUNX2, Collagen X, VEGFA, MMP-13, and a decrease in Collagen II expression, induced by IL-1β in SW1353 human chondrocytes. The potential molecular mechanism involved in the protective effects of WG was HDAC4 activation via the PTH1R/PKA/PP2A pathway. The inhibitory effect of WG was also associated with regulating the SOX9 and β-catenin signaling pathways. In vitro and in silico assessment suggested that 21 active compounds from licorice have the potential to bind stably with 11 targets (HDAC4, CTNNB1, COL10A1, MMP13, COL2A1, PRKACA, RUNX2, PTH1R, PTPA, SOX9, and VEGFA) related to chondrocyte hypertrophic change. Molecular docking analysis and previous in vivo studies suggest quercetin, glabridin, isoliquiritigenin, and kaempferol as major active compounds in licorice. Based on HPLC, isoliquiritigenin was identified as the major active compound in WG as having therapeutic effects on chondrocyte hypertrophy in OA cartilage. In addition, the metabolic precursors of quercetin, kaempferol, and isoliquiritigenin from WG may also have potential effects. These data reveal that WG and its major components will contribute to the development of new OA drugs that interfere with specific targets that are involved in chondrocyte hypertrophy.

## Figures and Tables

**Table 1:** ADME evaluation about 23 compounds by SwissADME.

Compound **	MW *	Consensus Log P	GI Absorption *	Bioavailability Score
**Quercetin**	302.24	1.23	High	0.55
**Pinocembrin**	256.25	2.26	High	0.55
**Liquiritigenin**	256.25	2.07	High	0.55
**Licoricidin**	424.53	5.05	High	0.55
**Licochalcone B**	286.28	2.14	High	0.55
**licochalcone A**	338.4	3.98	High	0.55
**Licobenzofuran**	354.4	4.15	High	0.55
**Licoagrochalcone A**	324.37	3.86	High	0.55
L-Aspartic Acid	133.1	−2.76	Low	0.56
**Kanzonol X**	394.5	5.06	High	0.55
**kanzonol W**	336.34	3.36	High	0.55
**kaempferol**	286.24	1.58	High	0.55
**Isoramanone**	348.48	2.25	High	0.55
**Isoliquiritigenin**	256.25	2.37	High	0.55
**Hispaglabridin B**	390.47	4.69	High	0.55
**Hispaglabridin A**	392.49	4.93	High	0.55
**Glabrone**	336.34	3.13	High	0.55
**Glabridin**	324.37	3.45	High	0.55
**Glabrene**	322.35	3.38	High	0.55
**Glabranin**	324.37	3.65	High	0.55
**Formononetin**	268.26	2.66	High	0.55
5-Hydroxyisophthalic Acid	182.13	0.54	High	0.56
**17-Hydroxy-11-Deoxy-Corticosterone**	360.49	3.02	High	0.55

MW *—molecular weight; GI absorption *—gastric intestinal absorption; Compound **—bolded compound indicates active compounds.

## Data Availability

Data is contained within the article and supplementary material.

## Supplementary Materials

The following are available online at https://www.mdpi.com/article/10.3390/ph14121337/s1. Table S1, Kaempferol and quercetin derivatives analysis of positive ES mode, Table S2, Kaempferol and quercetin derivatives analysis of negative ES mode, Table S3, Licorice-related compound/target information from TCMSP database, Table S4, Licorice-related compound/target information from BATMAN-TCM database (Score > 5), Table S5, 1803 Chondrocyte hypertrophy-related targets from GeneCards, Table S6, Gene enrichment analysis with Wikipathways of the 505 genes, Table S7, Endochondral ossification or Wnt signaling pathway-related genes, Table S8, Compounds and its number of genes which were associated with endochondral ossification or Wnt signaling pathway, Table S9, ADME evaluation about 23 compounds, Table S10, Docking energy between active compounds and each residues of HDAC4, CTNNB1, COL10A1, MMP13, COL2A1, PRKACA, RUNX2, PTH1R, PTPA, SOX9, VEGFA.
