# Supplementary material for: In Vitro Study of Licorice on IL-1β-Induced Chondrocytes and In Silico Approach for Osteoarthritis
Source: Pharmaceuticals (Basel). 2021 Dec 20;14(12):1337. doi: 10.3390/ph14121337 (PMC8709290; doi:10.3390/ph14121337)
Supplement: Supplementary file 1 [file pharmaceuticals-14-01337-s001.zip › Supplementary Materials and Methods.pdf]

Supplementary Materials and Methods

LC-QTOF-MS analysis

Unbiased metabolomics analysis was performed using an ultra-performance liquid chromatography (UPLC) system (Waters, Milford, USA). The chromatographic separation was carried out using an ACQUITY UPLC HSS T3 column (100 mm × 2.1 mm, 1.8 µm, Waters) with a column temperature of 40 °C and a flow rate of 0.5 ml/min, where the mobile phase contained solvent A (water +0.1% formic acid) and solvent B (acetonitrile +0.1% formic acid). Metabolites were eluted using the following gradient elution conditions: 97% phase A for 0–5 min; 3–100% liner gradient phase B for 5 ~16 min; 100% phase B for 16–17 min; 100–3% reverse liner gradient phase B for 17~19 min; 97% Phase A for 19–25 min. The loading volume of each sample was 5 µL. The metabolites eluted from the column were detected by a high-resolution tandem mass spectrometer SYNAPT G2 Si HDMS QTOF (Waters) in positive and negative ion modes. For positive ion mode, the capillary voltage and the cone voltage were set at 2 kV and 40 V, respectively. For negative ion mode, they were 1 kV and 40 V, respectively. Centroid MS^E mode was used to collect the mass spectrometry data. The primary scan ranged from 50 to 1200 Da and the scanning time was 0.2 s. All the parent ions were fragmented using 20–40 eV. The information of all fragments was collected and the time was 0.2 s. In the data acquisition process, the LE signal was gained every 3 s for real-time quality correction. For accurate mass acquisition, leucine enkephalin at a flow rate of 10 µL min^{-1} was used as a lock mass by a lock spray interface to monitor the positive ($[M + H]^+$ = 556.2771) and the negative ($[M - H]^-$ = 554.2615) ion modes. Data acquisition and analysis were controlled by Waters UNIFI V1.71 software. The scan rang in MS and MS/MS modes were over a range of 50–1200 m/z.
