# Supplementary material for: In Vitro Study of Licorice on IL-1β-Induced Chondrocytes and In Silico Approach for Osteoarthritis
Source: Pharmaceuticals (Basel). 2021 Dec 20;14(12):1337. doi: 10.3390/ph14121337 (PMC8709290; doi:10.3390/ph14121337)
Supplement: Supplementary file 1 [file pharmaceuticals-14-01337-s001.zip › Table_S1.pdf]

Table S1. Kaempferol and quercetin derivatives analysis of positive ES mode

| Component name | Neutral mass (Da) | Observed neutral mass (Da) | Observed m/z | Total Fragments Found | Mass error (mDa) | Mass error (ppm) | Observed RT (min) | Detector counts | Response | Adducts |
|---|---|---|---|---|---|---|---|---|---|---|
| 8-C-Prenylkaempferol | 354.1103 | 354.1113 | 355.1186 | 19 | 0.9 | 2.7 | 8.29 | 181862 | 150792 | +H |
| 5-Methyl kaempferol | 300.0634 | 300.0641 | 301.0713 | 13 | 0.7 | 2.2 | 6.45 | 57081 | 47251 | +H |
| Kaempferol | 286.0477 | 286.0481 | 287.0554 | 7 | 0.4 | 1.4 | 8.29 | 47583 | 40633 | +H |
| Kaempferol-7-O-α-L-rhamnoside | 432.1056 | 432.1067 | 433.1139 | 24 | 1 | 2.3 | 4.07 | 38044 | 28688 | +H |
| 5-Methyl kaempferol | 300.0634 | 300.064 | 301.0713 | 11 | 0.6 | 2.1 | 4.64 | 19800 | 17016 | +H |
| 5-Methyl kaempferol | 300.0634 | 300.0639 | 301.0712 | 44 | 0.5 | 1.8 | 7.36 | 18287 | 15881 | +H |
| 5-Methyl kaempferol | 300.0634 | 300.0639 | 301.0712 | 6 | 0.5 | 1.8 | 4.55 | 18102 | 12327 | +H |
| 5-Methyl kaempferol | 300.0634 | 300.064 | 301.0712 | 10 | 0.6 | 1.9 | 7.43 | 14867 | 12000 | +H |
| 8-C-Prenylkaempferol | 354.1103 | 354.111 | 355.1183 | 10 | 0.7 | 1.8 | 7.51 | 13759 | 5295 | +H |
| 8-C-Prenylkaempferol | 354.1103 | 354.1095 | 355.1168 | 15 | -0.8 | -2.3 | 6.3 | 7146 | 4791 | +H |
| 8-C-Prenylkaempferol | 354.1103 | 354.1112 | 355.1185 | 21 | 0.8 | 2.4 | 5.83 | 6763 | 4679 | +H |
| 8-C-Prenylkaempferol | 354.1103 | 354.112 | 355.1192 | 12 | 1.6 | 4.6 | 6.07 | 6049 | 4838 | +H |
| 8-C-Prenylkaempferol | 354.1103 | 354.11 | 355.1173 | 17 | -0.3 | -1 | 6.76 | 4915 | 3822 | +H |
| 8-C-Prenylkaempferol | 354.1103 | 354.1104 | 355.1176 | 21 | 0 | 0.1 | 5.98 | 3346 | 3346 | +H |
| Kaempferol | 286.0477 | 286.0487 | 287.0559 | 4 | 0.9 | 3.2 | 8.58 | 2719 | 2351 | +H |
| 8-C-Prenylkaempferol | 354.1103 | 354.1117 | 377.1009 | 33 | 1.4 | 3.7 | 10.19 | 912 | 685 | +Na |
| 8-C-Prenylkaempferol | 354.1103 | 354.1097 | 377.0989 | 35 | -0.7 | -1.7 | 8.47 | 789 | 789 | +Na |
| 5-Methyl kaempferol | 300.0634 | 300.0628 | 323.0521 | 10 | -0.5 | -1.7 | 9.08 | 725 | 725 | +Na |
| 8-C-Prenylkaempferol | 354.1103 | 354.1101 | 377.0993 | 79 | -0.2 | -0.6 | 8.05 | 691 | 691 | +Na |
| 8-C-Prenylkaempferol | 354.1103 | 354.1116 | 377.1008 | 18 | 1.2 | 3.2 | 7.04 | 599 | 599 | +Na |
| 8-C-Prenylkaempferol | 354.1103 | 354.1119 | 377.1011 | 7 | 1.6 | 4.2 | 8.95 | 521 | 521 | +Na |
| Quercetin-3,3'-dimethyl-ether | 330.074 | 330.0746 | 331.0818 | 7 | 0.6 | 1.9 | 7.31 | 23152 | 19058 | +H |
| Quercetin-3',4',7-trimethyl ether | 328.0947 | 328.0958 | 329.103 | 18 | 1.1 | 3.3 | 7.05 | 5364 | 4393 | +H |
| Quercetin 3,3'-dimethyl ether 7-rutinoside | 638.1847 | 638.1875 | 639.1948 | 14 | 2.9 | 4.5 | 6.73 | 3653 | 2079 | +H |
| Quercetin-3',4',7-trimethyl ether | 328.0947 | 328.0942 | 329.1014 | 18 | -0.5 | -1.6 | 8.02 | 2717 | 1650 | +H |
| Quercetin-3-O-β-D-xylopyranosyl (1→2)-β-D-glucopyranoside | 596.1377 | 596.1388 | 597.1461 | 0 | 1.1 | 1.9 | 15.64 | 1647 | 804 | +H |
| Quercetin-3',4',7-trimethyl ether | 328.0947 | 328.0953 | 329.1025 | 8 | 0.6 | 1.7 | 6.69 | 1310 | 726 | +H |
| Quercetin-7-O-rutinoside | 610.1534 | 610.151 | 611.1583 | 12 | -2.4 | -3.9 | 3.19 | 844 | 844 | +H |
| 3-O-β-D-Galacopyanosyl quercetin | 466.1111 | 466.1087 | 489.0979 | 27 | -2.4 | -4.9 | 0.59 | 526 | 526 | +Na |
