# Supplementary material for: In Vitro Study of Licorice on IL-1β-Induced Chondrocytes and In Silico Approach for Osteoarthritis
Source: Pharmaceuticals (Basel). 2021 Dec 20;14(12):1337. doi: 10.3390/ph14121337 (PMC8709290; doi:10.3390/ph14121337)
Supplement: Supplementary file 1 [file pharmaceuticals-14-01337-s001.zip › Table_S2.pdf]

Table S2. Kaempferol and quercetin derivatives analysis of negative ES mode

Component name	Identification status	Neutral mass (Da)	Observed neutral mass (Da)	Observed m/z	Mass error (mDa)	Mass error (ppm)	Observed RT (min)	Detector counts	Response	Adducts
Dihydrokaempferol-5-O-β-D-glucopyranoside	Identified	450.11621	450.1154	449.1081	-0.8	-1.8	4.39	217726	1690	-H
8-C-Prenylkaempferol	Identified	354.11034	354.1117	353.1045	1.4	4	9.28	171237	135154	-H
Kaempferol 3-Lathyroside	Identified	580.14282	580.1452	579.1379	2.4	4.1	6.18	134803	1543	-H
8-C-Prenylkaempferol	Identified	354.11034	354.1103	399.1085	0	0	5.78	125081	99034	+HCOO
7-O-β-D-Glucopyrano-syl-kaempferol	Identified	448.10056	448.1004	447.0931	-0.1	-0.3	4.22	86352	4198	-H
Dihydrokaempferol-5-O-β-D-glucopyranoside	Identified	450.11621	450.1159	449.1086	-0.3	-0.7	3.91	82813	28707	-H
8-C-Prenylkaempferol	Identified	354.11034	354.1118	353.1045	1.4	4.1	9.39	77811	57802	-H
Kaempferol-3-O-(2G-α-L-rhamnosyl)-rutinoside	Identified	740.21638	740.2151	739.2079	-1.2	-1.7	3.63	76180	18517	-H
8-C-Prenylkaempferol	Identified	354.11034	354.11	399.1082	-0.3	-0.8	5.5	73394	17399	+HCOO
8-C-Prenylkaempferol	Identified	354.11034	354.1117	353.1044	1.4	3.9	9.75	65674	65674	-H
Resokaempferol	Identified	270.05282	270.0534	269.0461	0.5	2	5.8	56153	37622	-H
Kaempferol 3-apioside-7-rhamnosyl-(1→6)-galactoside	Identified	726.20073	726.2015	725.1942	0.7	1	3.96	43132	12220	-H
5-Methyl kaempferol	Identified	300.06339	300.0639	299.0566	0.5	1.8	5.98	38257	30117	-H
Dihydrokaempferol-5-O-β-D-glucopyranoside	Identified	450.11621	450.1166	449.1093	0.4	0.9	3.65	36040	21538	-H
8-C-Prenylkaempferol	Identified	354.11034	354.1108	399.109	0.5	1.2	5.91	30575	9139	+HCOO
Kaempferol 3-apioside-7-rhamnosyl-(1→6)-galactoside	Identified	726.20073	726.1982	725.191	-2.5	-3.4	5.06	30546	5249	-H
Kaempferol 3-O-β-D-glucuronopyranosyl methyl ester	Identified	476.09548	476.0948	475.0875	-0.7	-1.4	3.53	27108	5460	-H
8-C-Prenylkaempferol	Identified	354.11034	354.1108	353.1036	0.5	1.4	8.16	26468	7645	-H
8-C-Prenylkaempferol	Identified	354.11034	354.1109	353.1036	0.5	1.5	8.04	25656	13225	-H
8-C-Prenylkaempferol	Identified	354.11034	354.111	399.1092	0.6	1.6	6.73	23195	13672	+HCOO
Kaempferol-3-Glucoside-3''-p-coumaroyl	Identified	580.15808	580.1594	579.1521	1.3	2.3	3.84	20847	13247	-H

Kaempferol-7-O-α-L-arabinofuranoside	Identified	418.09	418.0912	417.0839	1.2	2.9	3.92	19856	18406	-H
Dihydrokaempferol-5-O-β-D-glucopyranoside	Identified	450.11621	450.1177	449.1104	1.5	3.3	3.42	17576	3547	-H
8-C-Prenylkaempferol	Identified	354.11034	354.1102	399.1084	-0.1	-0.3	5.09	17195	7171	+HCOO
5-Methyl kaempferol	Identified	300.06339	300.0636	299.0563	0.2	0.8	6.32	15880	6794	-H
8-C-Prenylkaempferol	Identified	354.11034	354.1106	399.1088	0.2	0.6	6.23	15747	13713	+HCOO
8-C-Prenylkaempferol	Identified	354.11034	354.1099	353.1027	-0.4	-1.2	7.16	14607	6841	-H
8-C-Prenylkaempferol	Identified	354.11034	354.1094	399.1076	-0.9	-2.3	4.23	14127	10111	+HCOO
Kaempferol-3-glucuronide	Identified	464.09548	464.0947	463.0874	-0.8	-1.7	8.55	11747	976	-H
7-O-β-D-Glucopyrano-syl-kaempferol	Identified	448.10056	448.1018	447.0945	1.2	2.8	4.02	9188	4477	-H
Kaempferol-3-Glucoside-3''-p-coumaroyl	Identified	580.15808	580.16	625.1582	1.9	3.1	2.58	8924	3732	+HCOO
Dihydrokaempferol-5-O-β-D-glucopyranoside	Identified	450.11621	450.1174	449.1102	1.2	2.7	5	6082	4356	-H
7-α-L-Rhamnosyl kaempferol 3-O-β-D-glucopyranosyl(1→6)-β-D-glucopyranoside	Identified	756.21129	756.2127	755.2054	1.4	1.9	10.24	4036	1693	-H
Kaempferol	Identified	286.04774	286.0465	285.0392	-1.3	-4.4	4.75	3921	3219	-H
7-α-L-Rhamnosyl kaempferol 3-O-β-D-glucopyranosyl(1→6)-β-D-glucopyranoside	Identified	756.21129	756.2124	755.2052	1.1	1.5	8.05	2808	1173	-H
Dihydrokaempferol-5-O-β-D-glucopyranoside	Identified	450.11621	450.1148	449.1075	-1.5	-3.2	6.7	2726	1295	-H
Dihydrokaempferol	Identified	288.06339	288.0621	287.0548	-1.3	-4.6	5.98	2557	2125	-H
Kaempferol-3-O-(2G-α-L-rhamnosyl)-rutinoside	Identified	740.21638	740.2188	739.2115	2.4	3.2	7.69	1880	1880	-H
Kaempferol-3-Glucoside-2''-p-coumaroyl	Identified	594.13734	594.1364	639.1346	-0.9	-1.4	6.27	1380	1380	+HCOO
Quercetin 3,3'-dimethyl ether 7-rutinoside	Identified	638.18469	638.184	637.1767	-0.7	-1.1	5	662025	11556	-H
Quercetin-3-O-(2G-α-L-rhamnosyl)-rutinoside	Identified	756.21129	756.2126	755.2054	1.3	1.8	3.41	132720	11096	-H

Quercetin 3,3'-dimethyl ether 7-rutinoside	Identified	638.18469	638.1864	683.1846	1.7	2.5	5.42	106550	4438	+HCOO
Quercetin 3,3'-dimethyl ether 7-rutinoside	Identified	638.18469	638.1853	637.178	0.6	1	5.16	103403	4692	-H
Quercetin 3,3'-dimethyl ether 7-rutinoside	Identified	638.18469	638.1842	637.1769	-0.5	-0.8	5.56	59327	3191	-H
Quercetin 3,3'-dimethyl ether 7-rutinoside	Identified	638.18469	638.1848	637.1775	0.1	0.2	4.87	32299	5944	-H
Quercetin 3,3'-dimethyl ether 7-rutinoside	Identified	638.18469	638.1842	683.1824	-0.5	-0.7	5.63	28299	4883	+HCOO
Quercetin 3,3'-dimethyl ether 7-rutinoside	Identified	638.18469	638.1868	683.185	2.1	3.1	2.7	22976	6705	+HCOO
Quercetin-7-O-rutinoside	Identified	610.15338	610.1539	609.1467	0.5	0.9	4.45	13307	7495	-H
Quercetin 3-O-neohesperidoside	Identified	610.15338	610.1564	609.1491	3	5	3.55	12035	7526	-H
Quercetin 3,3'-dimethyl ether 7-rutinoside	Identified	638.18469	638.1821	637.1748	-2.6	-4.1	4.45	5716	3674	-H
Quercetin-3-O-(2G-α-L-rhamnosyl)-rutinoside	Identified	756.21129	756.2079	801.2061	-3.4	-4.3	9.14	1219	1219	+HCOO
